# Intestinal Permeability, Gut Inflammation, and Gut Immune System Response Are Linked to Aging-Related Changes in Gut Microbiota Composition: A Study in Female Mice

**DOI:** 10.1093/gerona/glae045

**Published:** 2024-02-14

**Authors:** Paola Elizabeth Gámez-Macías, Elisa Félix-Soriano, Mirian Samblas, Neira Sáinz, María Jesús Moreno-Aliaga, Pedro González-Muniesa

**Affiliations:** Faculty of Pharmacy and Nutrition, Department of Nutrition, Food Science, and Physiology, University of Navarra, Pamplona, Spain; Center for Nutrition Research, University of Navarra, Pamplona, Spain; Faculty of Pharmacy and Nutrition, Department of Nutrition, Food Science, and Physiology, University of Navarra, Pamplona, Spain; Center for Nutrition Research, University of Navarra, Pamplona, Spain; Center for Nutrition Research, University of Navarra, Pamplona, Spain; Center for Nutrition Research, University of Navarra, Pamplona, Spain; Faculty of Pharmacy and Nutrition, Department of Nutrition, Food Science, and Physiology, and Center for Nutrition Research, University of Navarra/Navarra Institute for Health Research (IdiSNA), Pamplona, Spain; Centro de Investigación Biomédica en Red de Fisiopatología de la Obesidad y Nutrición, Instituto de Salud Carlos III, Madrid, Spain; Faculty of Pharmacy and Nutrition, Department of Nutrition, Food Science, and Physiology, and Center for Nutrition Research, University of Navarra/Navarra Institute for Health Research (IdiSNA), Pamplona, Spain; Centro de Investigación Biomédica en Red de Fisiopatología de la Obesidad y Nutrición, Instituto de Salud Carlos III, Madrid, Spain; (Biological Sciences Section)

**Keywords:** Colon, Diversity, Gut microbiota, Immune response, Intestinal barrier

## Abstract

Aging entails changes at the cellular level that increase the risk of various pathologies. An association between gut microbiota and age-related diseases has also been attributed. This study aims to analyze changes in fecal microbiota composition and their association with genes related to immune response, gut inflammation, and intestinal barrier impairment. Fecal samples of female mice at different ages (2 months, 6 months, 12 months, and 18 months) and gene expression in colon tissue were analyzed. Results showed that the older mice group had a more diverse microbiota than the younger group. Additionally, the abundance of *Cyanobacteria, Proteobacteria, Flavobacteriaceae, Bacteroides, Parabacteroides, Prevotellaceae_UCG-001, Akkermansia,* and *Parabacteroides goldsteinii* increased with age. In contrast, there was a notable decline in *Clostridiaceae, Lactobacillaceae, Monoglobaceae, Ligilactobacillus, Limosilactobacillus, Mucispirillum*, and *Bacteroides faecichinchillae*. These bacteria imbalances were positively correlated with increased inflammation markers in the colon, including *Tnf-α, Ccl2*, and *Ccl12*, and negatively with the expression of tight junction genes (*Jam2, Tjp1*, and *Tjp2*), as well as immune response genes (*Cd4, Cd72, Tlr7, Tlr12*, and *Lbp*). In conclusion, high levels of diversity did not result in improved health in older mice; however, the imbalance in bacteria abundance that occurs with aging might contribute to immune senescence, inflammation, and leaky gut disease.

The proportion of older people around the world is increasing dramatically. Several diseases are associated with the process of aging as a result of the accumulation of a number of molecular and cellular damages (1). For example, as we age, the intestine, which plays a critical role in immunity and acts as a barrier for pathogens and toxins, decreases its ability to self-repair after damage resulting in a leaky gut (2).

It has been widely recognized that the intestinal barrier has a direct impact on health and disease. The intestinal epithelium is a single-cell layer connected by intercellular tight junction proteins such as claudins, junctional adhesion molecules, and zonula occludens. These proteins are downregulated by inflammatory cytokines, thus increasing the risk of endotoxemia and causing inflammation on a local and systemic level (3–5). In addition, inflammation and intestinal permeability have been linked with an imbalance of intestinal bacteria (dysbiosis) (6,7). A recent study in obese older mice showed that *Lactobacillus* and *Enterococcus* strains may contribute to decreasing gut inflammation and enhancing the expression of tight junctions (8). Physiological changes related to aging may have an impact on gut microbiota composition and diversity (9). Currently, the research on this topic is limited, and there are conflicting results regarding the association between aging and some bacteria because both an increase (10,11) and a decrease (4) have been observed in the same species. It has been shown, for example, that although some studies suggest *Akkermansia* abundance increases with age (12,13), others have found the exact opposite (10,14). There is evidence that this bacterium plays a significant role in improving immunomodulation, reducing inflammation, and maintaining gut barrier function (10,14). Interestingly, it has also been found that centenarians have higher levels of this bacteria than healthy adults (10).

The aim of this study was to analyze changes in fecal microbiota composition and their association with genes related to immune response, gut inflammation, and intestinal barrier impairment. We investigated fecal microbiota composition at different lifespan states: young (2 months old), adult (6 months old), middle-aged (12 months old), and old (18 months old). To reach this objective, samples from the OBELEX project were used. As part of this study, female mice were studied and our team found metabolic disorders including an increase in glucose and cholesterol levels, as well as changes in body composition such as an increase in visceral fat levels as the mice aged (15).

## Materials and Methods

### Mice and Study Design

C57BL/6J female mice (Harlan Laboratories, Barcelona, Spain) were housed at the animal facilities of the University of Navarra in a specific pathogen-free environment under controlled conditions (22 ± 2°C, with a 12-hour light-dark cycle, relative humidity, 55 ± 10%). In total, 37 mice were fed ad libitum with a normal diet containing: 20% proteins, 67% carbohydrates, and 13% lipids (Harlan Teklad Global Diets, Harlan Laboratories, Indianapolis, IN, USA), and with free access to water. Mice were sacrificed at different periods: 2 months old (*n* = 10), 6 months old (*n* = 7), 12 months old (*n* = 10), and 18 months old (*n* = 10), and tissues and feces were collected and immediately stored at −80°C. It is important to note that this study is part of the OBELEX project of the University of Navarra, thus 3 mice from the 6-month-old group were sacrificed to obtain samples for other analyses. All experiments were performed by national animal care guidelines, and with the approval of the Ethics Committee for Animal Experimentation of the University of Navarra (Protocol 113-15), under the EU Directive *2010/63/EU.*

### cDNA Synthesis and Quantitative Real-Time PCR

RNA was isolated from colon tissue using TRIzol Reagent (Invitrogen, ThermoFisher Scientific, Waltham, MA, USA). RNA (1–5 g) was then incubated with DNase I (RapidOut DNA Removal kit, Thermo Fisher Scientific) at 37°C for 30 minutes. RNA quality and concentration were determined using the Nanodrop Spectrophotometer ND1000 (Nanodrop Technologies, Inc., Wilmington, NC, USA). Retrotranscription to cDNA was performed using High-Capacity cDNA Reverse Transcription (Applied Biosystems, Foster City, CA, USA) according to the manufacturer’s instructions. Then, quantitative PCR was performed using the Touch Real-Time PCR System (C1000 + CFX384, BIO-RAD, Hercules, CA, USA), and gene expression was analyzed using Power SYBR Green PCR Master Mix. Primers were obtained from the online PrimerBank and a previous study (16) (Supplementary Table S1). Relative gene expression was determined by the 2^−ΔΔCT^ method, and the expression data were normalized with the *Gapdh* gene expression. The samples were processed in duplicate, and the mean values were used for statistical analysis.

### Fecal DNA Extraction and 16S rRNA Gene Sequencing

Microbiota analysis in stool samples was performed in collaboration with CIMA LAB Diagnostics (University of Navarra), which followed the established protocol. Briefly, the Maxwell RSC Fecal Microbiome DNA kit (Promega, Madison, WI, USA) in the Maxwell RSC Instrument was used to efficiently isolate DNA from OMNIgene. GUT kits following the manufacturer’s instructions; dsDNA characterization was performed with Qubit (ThermoFisher Scientific). Two PCR reaction protocols were used to prepare samples for sequencing the variable V3 and V4 regions of the 16S rRNA gene. The first PCR was performed with the next instructions: (1) 50 ng of dsDNA in 2.5 µL, (2) 15.5 µL of master mix, (3) 5 µL of F and 5 µL R primers (Forward Primer: 5ʹ TCGTCGGCAGCGTCAGATGTGTATAAGAGACAGCCTACGGGNGGCWGCAG; Reverse Primer: 5ʹ GTCTCGTGGGCTCGGAGATGTGTATAAGAGACAGGACTACHVGGGTATCTAATCC), and (4) the following PCR program within SimpliAmp Thermal Cycler (ThermoFisher Scientific): 95°C for 3 minutes, and 25 cycles of 95°C for 30 seconds, 55°C for 30 seconds, 72°C for 30 seconds, and finally, 72°C for 5 minutes, to later keep refrigerated at 4°C. The PCR product was purified using AMPURE and quantified using Qubit (ThermoFisher Scientific).

The second PCR was performed following the next instructions: (1) 15 µL of 1st PCR product, (2) 25 µL of master mix, (3) 5 µL of F and 5 µL of R primers (indexing step), and (4) the following PCR program within SimpliAmp Thermal Cycler (ThermoFisher Scientific): 95°C for 3 minutes, and 8 cycles of 95°C for 30 seconds, 55°C for 30 seconds, 72°C for 30 seconds, and finally, 72°C for 5 minutes, to later keep refrigerated at 4°C. The PCR product was purified using AMPURE and quantified using Qubit (ThermoFisher Scientific). The sequencing was performed on the Illumina MiSeq SY-410-1003 system according to the manufacturer’s protocols.

### Bioinformatics and Statistical Analysis

Bioinformatics analyses were performed in collaboration with the Bioinformatics Platform of CIMA (University of Navarra). 16S rRNA sequences obtained were filtered following quality criteria of the OTU processing pipeline LotuS2 (17) (release 2.19). This pipeline includes UPARSE de novo sequence clustering (18), removal of chimeric sequences and phix contaminants (19) for the identification of OTUs (Operational Taxonomic Units), and OTU abundance matrix generation. Finally, taxonomy was assigned using lambda aligner (20) and SILVA 16S/18S database (21) achieving up to species-level sensitivity.

Data raw counts were normalized (center log-ratio) with the *ALDEx* R-script package. Shannon and Simpson’s indexes were calculated at the genus level using the *microbiome* R-script package to determine the alpha diversity. Beta diversity was determined and visualized in a non-metric multidimensional scaling (NMDS) plot based on Bray–Curtis distances at the OTUs level using the *ecodist* R-script package. For multiple comparisons, bacteria not found in at least half the samples from each group were discarded. Relative abundance was used for the graphical representation. Kruskal–Wallis’s test and the post hoc Bonferroni test were used to adjust multiple comparisons.

For genes, the one-way ANOVA test was used when the data were normally distributed. Kruskal–Wallis’s test was used when the data were not normal. The Tukey (parametric) and Tamhane (nonparametric) tests post hoc were performed to determine differences between groups. Associations between relative abundance and gene expression were calculated using Spearman’s rank correlation coefficient. The *p* value < .05 was considered statistically significant and correlations were adjusted by Benjamini–Hochberg’s FDR controlling procedure (*q* value < 0.05).

The analyses were performed using RStudio 2022.07.1 for macOS, and gene graphs were performed with GraphPad Prism 9.1.2 for macOS. Data are shown as means ± SEM unless otherwise noted.

## Results

### Diversity and Composition

**Figure 1.**
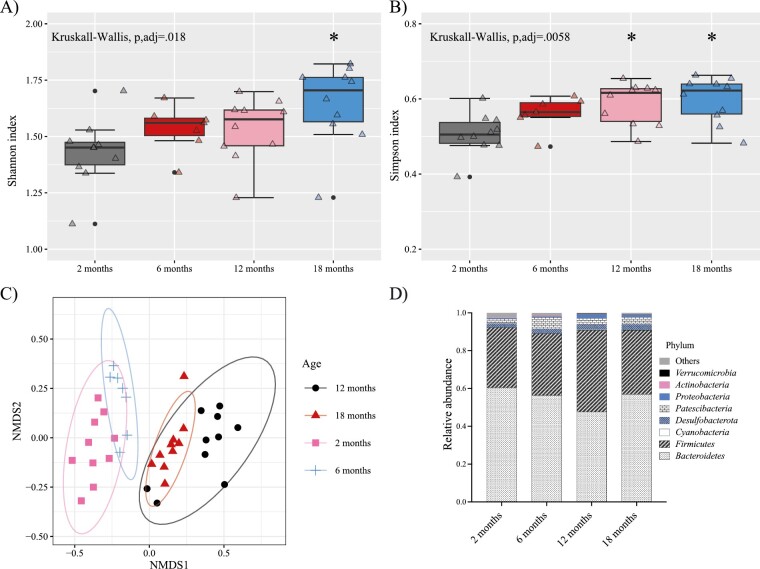
Comparative analysis of alpha diversity at genus level in young (2 months), adult (6 months), middle-aged (12 months), and old (18 months) female mice: (A) Shannon index and (B) Simpson index. Data are presented as mean ± SEM; (C) non-metric multidimensional scaling (NMDS) at the OTUS level based on Bray–Curtis distances showing dissimilarities between groups. (D) Relative abundance at the phylum level. *p < .05 versus 2 months. OTUS = operational taxonomic units; SEM = standard error of the mean.

To determine how age affects the gut microbiota, we first evaluated differences in alpha (a summary of the microbial community richness and evenness in each sample (22)) and beta diversity (a measure of interindividual diversity assessing the similarity between communities (22)) at 2, 6, 12, and 18 months of age. According to the Shannon and Simpson indexes, alpha diversity significantly increased as the mice aged, as shown in Figure 1A and B. Beta diversity was analyzed by NMDS based on Bray–Curtis distances at the OTUS level, which revealed that age clusters are different (Figure 1C).

**Figure 2.**
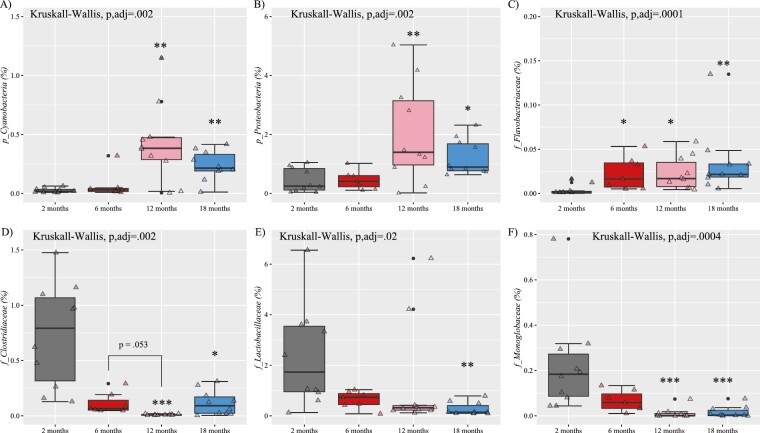
Relative abundance of some phyla (A and B) and families (C–F) differs by age in female mice. Data are presented as mean ± SEM. f = family, p = phyla. ***p < .001, **p < .01, *p < .05 versus 2 months. SEM = standard error of the mean.

Next, different taxonomic levels were analyzed (phylum, family, genus, and species). As detailed in Figure 1D  *Bacteroidetes* and *Firmicutes* were the most abundant phyla in all groups. Furthermore, the results of the study indicated that older groups had a significantly increased abundance of the phylum *Cyanobacteria* and *Proteobacteria* (Figure 2A and B). In addition, the abundance of the *Flavobacteriaceae* family, well-known pathogens (23), increased from 6 months old onwards (Figure 2C). In contrast, there was a notable decline in the *Clostridiaceae*, *Lactobacillaceae,* and *Monoglobaceae* families, members of the *Firmicutes* phylum (Figure 2D–F).

**Figure 3.**
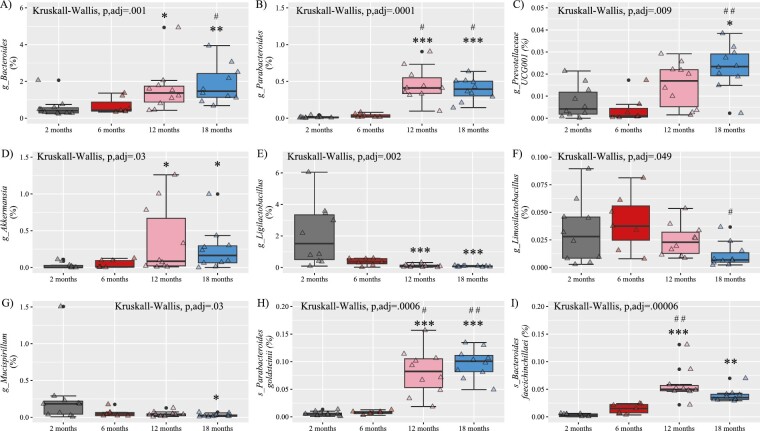
Relative abundance of the main age-related genus (A–G) and species (H–I) shifts in female mice. Data are presented as mean ± SEM. g = genus; s = species. *p < .05, **p < .01, ***p < .001 versus 2 months; ^#^*p* < .05, ^##^*p* < .01 versus 6 months. SEM = standard error of the mean.

At the genus level, aging promoted higher levels of *Bacteroides, Parabacteroides, Prevotellaceae_UCG-001*, and *Akkermansia* (Figure 3A–D). On the contrary, aging was accompanied by lower levels of *Ligilactobacillus, Limosilactobacillus*, and *Mucispirillum* (Figure 3E–G). Finally, at the species level, we found an unexpected increase in *Parabacteroides goldsteinii* in middle-aged and old mice (Figure 3H), which has been associated with improving intestinal integrity and reducing inflammation (24). Additionally, we found an increase, as mice aged, in the abundance of *Bacteroides faecichinchillae*, which has been poorly studied (Figure 3I).

### Gut Permeability

**Figure 4.**
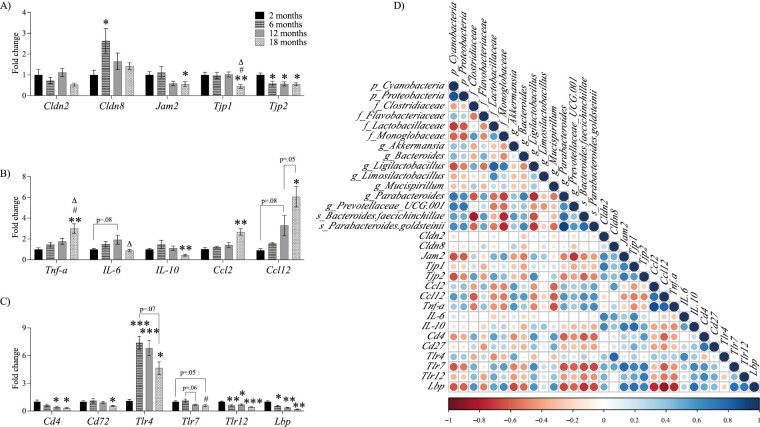
Age-related changes in the expression of genes related to (A) intestinal barrier function, (B) inflammatory marker genes, and (C) immunity genes assessed in colon tissue of female mice. Data are presented as mean ± SEM. *p < .05, **p < .01, ***p < .001 versus 2 months; ^#^*p* < .05 versus 6 months; ^Δ^*p* < .05 versus 12 months. (D) Correlation plots for the gene expression represent the Spearman coefficient in positive (blue) and negative (red) colors, and the larger the circle, the stronger the correlation. f = family, g = genus, p = phyla, s = species; SEM = standard error of the mean.

We estimated potential changes in gut permeability through the analysis of the expression of different tight junction genes. Intestinal epithelial cells are connected by tight junctions (claudins, junctional adhesion molecules, and zonula occludens), which maintain intestinal barrier integrity (3). Age-related changes are evident in our results (Figure 4A). Claudin 8 (*Cldn8)* increased significantly between 2 months and 6 months (*p* = .04). The junctional adhesion molecule 2 (*Jam2*) decreased as the mice aged, as observed when comparing 2 months versus 18-month-old mice (*p* = .04). The tight junction protein 1 (*Tjp1*) remains stable in adult and middle-aged mice but exhibited a significant downregulation in old mice (2 months vs 18 months, *p* = .008). However, the tight junction protein 2 (*Tjp2*) was downregulated earlier (2 months vs 6 months, *p* = .01) and remained at this level until 18 months old (*p* = .01).

The Spearman correlation coefficient was calculated to find associations between bacterial abundance and gene expression (Figure 4D). We found a positive correlation between *Jam2* gene expression and bacteria that were present in high levels in younger mice compared with older mice, such as the *Lactobacillaceae* (rho = 0.4, *p* = .03) and *Monoglobaceae* families (rho = 0.5, *p* = .001), as well as the *Ligilactobacillus* genus (rho = 0.4, *p* = .03). Moreover, the following taxa were present in low percentages in the younger groups and there was a negative correlation between *Jam2* and them: *Cyanobacteria* (rho = −0.6, *p* = .001), *Proteobacteria* (rho = −0.6, *p* = .002), *Akkermansia* (rho = −0.4, *p* = .04), *Bacteroides* (rho = −0.5, *p* = .01), *Parabacteroides* (rho = −0.5, *p* = .01), *Prevotellaceae_UCG-001* (rho = −0.7, *p* < .001), *B faecichinchillae* (rho = −0.5, *p* = .01), and *P goldsteinii* (rho = −0.4, *p* = .049). Regarding *Tjp1*, we found only a negative correlation with *Bacteroides* increase (rho = −0.4, *p* = .04). There were, however, statistically significant positive correlations between *Tjp2* and high levels of the *Clostridiaceae* (rho = 0.5, *p* = .005), *Lactobacillaceae* (rho = 0.5, *p* = .005), and *Monoglobaceae* families (rho = 0.4, *p* = .03), and the *Ligilactobacillus* genus (rho = 0.6, *p* < .001). Further, the results indicate that *Tjp2* was negatively correlated with several bacteria that increased as mice aged, including *Cyanobacteria* (rho = −0.6, *p* = .001), *Proteobacteria* (rho = −0.5, *p* = .01), *Flavobacteriaceae* (rho = −0.04, *p* = .03), *Bacteroides* (rho = −0.4, *p* = .02), *Parabacteroides* (rho = −0.5, *p* = .01), *Prevotellaceae_UCG-001* (rho = −0.4, *p* = .04), *B faecichinchillae* (rho = −0.6, *p* = .002), and *P goldsteinii* (rho = 0.4, *p* = .01).

### Gut Inflammation

Afterward, some inflammation markers in colon tissue were assessed (Figure 4B). There was an increase in *Tnf-α* expression as the mice grew older (2 months vs 18 months, *p* = .001). Several studies have suggested that excessive production of proinflammatory cytokines, such as TNF-*α*, may contribute to the increase in intestinal permeability by modulating tight junction proteins (3,4,9), which is consistent with the correlation between *Tnf-α* and *Tjp1* found in this study (rho = −0.5, *p* = .02), as shown in Figure 4D. In contrast, IL-6 plays a key role in maintaining intestinal epithelial development and homeostasis. It has been suggested that IL-6 stimulates intestinal epithelial cells to secrete claudin-2 (25). In the present study, mice showed a significant decline in *IL-6* expression between 12 months and 18 months of age (*p* = .04). In addition, there was a positive correlation between *IL-6* and *Cldn2* (rho = 0.6, *p* < .001), see Figure 4D. Similarly, IL-10 upregulates the expression of tight junction proteins by suppressing proinflammatory cytokine secretion (26). In the current study, *Il-10* expression was reduced in older mice (2 months vs 18 months, *p* = .001). Moreover, *Il-10* was positively correlated with tight junction gene expression such as *Cldn2* (rho = 0.6, *p* = .02), *Jam2* (rho = 0.6, *p* = .01), and *Tjp1* (rho = 0.7, *p* = .004), see Figure 4D. On the other hand, CCL2 and its homolog CCL12 have been implicated in inflammatory conditions, and high levels of CCL2 have been linked to shortening life expectancy (27). We found a marked increase with aging in the expression of both *Ccl2* and *Ccl12* genes (2 months vs 18 months, *p* = .003 and *p* = .03, respectively).

Furthermore, we searched for associations between these inflammatory genes and bacteria. As detailed in Figure 4D, *Tnf-α* was positively correlated with an increase in the proportion of the *Proteobacteria* phylum (rho = 0.4, *p* = .02), the *Flavobacteriaceae* family (rho = 0.4, *p* = .049), *Akkermansia* (rho = 0.5, *p* = .003), and *Parabacteroides* genus (rho = 0.5, *p* = .01); and the species *B* (rho = 0.5, *p* = .004), and *P goldsteinii* (rho = 0.6, *p* = .001). Conversely, the low abundance of *Clostridiaceae* (rho = −0.5, *p* = .01), *Lactobacillaceae* (rho = −0.4, *p* = .03), and *Monoglobaceae* families (rho = −0.5, *p* = .01) exhibited a negative association with *Tnf-α* as well as the *Ligilactobacillus* (rho = −0.5, *p* = .01), and *Mucispirillum* genus (rho = −0.6, *p* < .001). Additionally, a positive correlation was found between the chemokine *Ccl2* and high levels found in older groups of *Akkermansia* (rho = 0.5, *p* = .01) as well as *P goldsteinii* (rho = 0.5, *p* = .01). By contrast, a negative correlation was found with the decline in the abundance of *Lactobacillaceae* (rho = −0.4, *p* = .03), *Monoglobaceae* families (rho = −0.5, *p* = .01), and *Ligilactobacillus* (rho = −0.5, *p* = .01), and *Mucispirillum* genus (rho = −0.6, *p* < .001). Significant correlations were not found between age-related changes in bacterial abundance and the gene expression of the antiinflammatory cytokine *Il-10*.

### Immune Response

Lastly, some genes were analyzed to assess the gut immune response (Figure 4C). It has been proposed that gut microbiota regulate intestinal immunity, and the immune system can also be influenced by age (5). A total of 6 immune genes were studied from colon tissue. The expression of *Cd4*, which is required for initiation or enhancement of T-cell activation (28), was downregulated in older mice (*p* = .02). Similarly, Cd72 which plays a role in B-cell proliferation and differentiation (29) was decreased in the older group (*p* = .02). Moreover, toll-like receptors (*Tlr*) have a key role in pathogen recognition and innate immune response by activating inflammatory signaling pathways, stimulating immunoglobulin A (IgA) production, maintaining tight junctions, and promoting antimicrobial peptide expression (30). There was a significant increase in the *Tlr4* gene expression in adult mice (2 months vs 6 months, *p* < .001), and it remained high in middle-aged and older mice. Interestingly, this study showed that *Tlr7* expression was downregulated from 6 to 18 months (*p* = .041), as well as *Tlr12* from 2 months to 18 months (*p* < .001). Finally, the lipopolysaccharide-binding protein (LBP) enhances the recognition of endotoxin and bacterial surfaces by the immune system (31). According to the results of this study, *Lbp* downregulation in mice began at 6 months of age (2 months vs 6 months, *p* < .04).

A correlation analysis was conducted, as before, to determine a relationship between immunity gene expression and microbiota composition (Figure 4D). Significant positive correlations were found between *Cd4* and the bacteria that diminish with aging such as *Clostridiaceae* (rho = 0.5, *p* = .02), and *Lactobacillaceae* families (rho = 0.5, *p* = .007), as well as *Ligilactobacillus* (rho = 0.6, *p* = .003), and *Mucispirillum* genus (rho = 0.6, *p* = .005). In contrast, those that increased in the older mice showed a negative correlation with *Cd4* gene expression: *Cyanobacteria* (rho = −0.4, *p* = .02), *Akkermansia* (rho = −0.5, *p* = .02), *Parabacteroides* (rho = −0.6, *p* = .002), *Prevotellaceae_UCG-001* (rho = −0.4, *p* = .02), *B faecichinchillae* (rho = −0.6, *p* = .001), *P goldsteinii* (rho = −0.5, *p* = .003). Regarding *Cd72*, there was a positive correlation with the increase in the *Mucispirillum* genus (rho = 0.5, *p* = .02), and a negative correlation with low levels of *Flavobacteriaceae* family (rho = −0.4, p = .03). Concerning the *Tlr7* gene, bacteria that in young mice groups were more abundant compared to the older group and had a positive correlation with the *Clostridiaceae* (rho = 0.5, *p* = .05), *Lactobacillaceae* (rho = 0.5, *p* = .01), and *Monoglobaceae* (rho = 0.6, *p* = .005) families, and the *Ligilactobacillus* genus (rho = 0.6, *p* = .002). On the contrary, bacteria increased in older mice such as the *Cyanobacteria* (rho = −0.6, *p* = .001), *Proteobacteria* (rho = −0.5, *p* = .009), *Akkermansia* (rho = −0.5, *p* = .01), *Bacteroides* (rho = −0.4, *p* = .03), *Parabacteroides* (rho = −0.6, *p* < .001), *Prevotellaceae_UCG-001* (rho = −0.6, *p* = .002), *B faecichinchillae* (rho = −0.6, *p* = .001), and *P goldsteinii* (rho = −0.6, *p* < .001) showed a negative correlation with the *Tlr7* mRNA expression. The *Tlr12* expression was positively correlated with bacteria that were most abundant among young mice groups including the families *Clostridiaceae* (rho = 0.4, *p* = .03), *Lactobacillaceae* (rho = 0.5, *p* = .01), and *Monoglobaceae* (rho = 0.5, *p* = .005). On the contrary, *Tlr12* was negatively correlated with bacteria that have distinguished aging in mice: the *Flavobacteriaceae* family (rho = −0.4, *p* = .04), the *Akkermansia* (rho = −0.4, *p* = .03), and *Parabacteroides* genus (rho = −0.4, *p* = .03), as well as the *B faecichinchillae* (rho = −0.5, *p* = .007), and *P goldsteinii* (rho = −0.4, *p* = .03) species. Finally, *Lbp* gene expression was positively correlated with bacteria that declined as mice age: family *Clostridiaceae* (rho = 0.6, *p* = .006), *Lactobacillaceae* (rho = 0.5, *p* = .01), and *Monoglobaceae* (rho = 0.6, *p* = .001), as well as the genus *Ligilactobacillus* (rho = 0.6, *p* = .005), and *Mucispirillum* (rho = 0.6, *p* = .004); and negatively correlated with bacteria found in younger mice at low levels: *Cyanobacteria* (rho = −0.6, *p* = .002), and *Proteobacteria* (rho = −0.6, *p* = .005) phyla, the *Flavobacteriaceae* family (rho = −0.5, *p* = .01), the genus *Akkermansia* (rho = −0.6, *p* = .001), *Bacteroides* (rho = −0.6, *p* = .006), *Parabacteroides* (rho = −0.7, *p* < .001), *Prevotellaceae_UCG-001* (rho = −0.6, *p* = .004), and species such as *P goldsteinii* (rho = −0.6, *p* = .001), and *B faecichinchillae* (rho = −0.7, *p* < .001).

## Discussion

Gut microbiota composition could be considered a hallmark of aging (32). Although numerous studies in this field have been conducted, gut microbiota composition in aging as well as their relationship to age-related health decline remain poorly understood (33,34). We examined the gut microbiota composition of female mice in 4 age groups (young, adult, middle-aged, and old) and their association with markers of intestinal permeability, colon inflammation, and intestinal immune response. Results of this study indicate that the older group had a more diverse microbiota than the younger groups, which agrees with other studies conducted on male mice (4,13,35,36). Unfortunately, studies in female mice have been limited on this topic. We found a study conducted by Cao et al., in female mice, where a reduction in bacterial diversity was observed, but their diet had a higher carbohydrate content compared with our study. Evaluating the effect of the impact of the diet has not been the objective of the present study, the animals were fed with a standard diet. Based on this, it is possible that a healthy diet could prevent bacterial diversity decline in aged mice. Further research is needed to assess the impact of different diets on aging and gut microbiota composition and its link to healthy aging (12).

In our study, the abundance of pathogens such as the *Cyanobacteria* and *Proteobacteria* phyla increased with aging. A study conducted by Bárcena et al. (10) found similar results using mouse models of progeria (a genetic disorder that speeds up aging) in both sexes, as well as the study of Liu et al. (4) with humans and mice samples. Furthermore, we found the abundance of these bacteria was positively correlated with gut inflammation, a weak gut barrier, and a diminished immune response. *Cyanobacteria* produce toxins that lead to neurodegenerative diseases and have also been linked to irritable bowel disease (37). To our knowledge, this is the first time that their relationship with intestinal permeability and weak immune response was observed, which points to the importance of conducting further research to understand the role of this phylum in human health. Many bacteria that belong to the *Proteobacteria* phylum are pathogenic to humans as well (38). Hexaacylated form of lipopolysaccharide produced by these bacteria may cause chronic inflammation (39) a characteristic of aging that could be related to the colon inflammation observed in our study.

Furthermore, we found a higher abundance in old mice of the *Flavobacteriaceae* family, the *Bacteroides, Parabacteroides,* and *Prevotellaceae_UGC-100* genus, and the *P goldsteinii*, and *B faecichinchillae* species, which are members of the *Bacteroidetes* phylum. They were all negatively correlated with the expression of genes related to immunity. A recent study by Qiu et al. (40) showed a positive correlation between *Flavobacteriales* and immunosuppressive biomarkers in zebrafish. However, the *Flavobacteriaceae* family has not been extensively studied, thus more research is necessary to clarify its role. According to our knowledge, this is the first time this family has been linked to aging and intestinal permeability, thus the increase of these bacteria in old mice could influence the expression of *Tjp2* in the colon.

Regarding *Bacteroides*, they are gram-negative bacteria and can modulate their cell-wall polysaccharides to alter their immunity recognition capabilities (41). Therefore, this genus is able to avoid the immune response of the host, which explains the negative correlation with the immunity-related genes found in this study. In a recent study, supplementation with a *Parabacteroides* strain prevented the disruption of tight junction proteins by inhibiting NF-Kβ activation and lowering inflammation levels (42). Additionally, *P goldsteinii* produces acetic and succinic acids as end-products of glucose metabolism and has been negatively correlated with liver inflammation (43,44). In contrast, in the present study, this bacterium was found in high abundance in old mice, also the behavior of this bacteria showed a link with the inflammation markers and with a decrease in tight junction expression; therefore, the increase in the presence of this strain might be related to the intestinal disease of aging. The study led by Guo et al. (45) also reported significantly higher levels of the *Parabacteroides* genus in frail older persons suggesting that this genus may play a crucial role in the development of diseases related to aging. Continuing research on this bacterium could potentially serve as an indirect marker for evaluating health status in aging.

In contrast, we observed that the abundance of the *Clostridiaceae*, *Lactobacillaceae*, and *Monoglobaceae* families, as well as the *Ligilactobacillus* genus, were reduced in older mice. Interestingly, the present study also suggests that as mice age, their tight junction expression decreases, and there is a link between this and the decrease in the *Clostridiaceae* family which could be explained by the production of the metabolites of these bacteria. It is widely known that *Clostridium* bacteria are butyrate producers, according to the study by Fang et al. (46), butyrate enhances the expression of tight junctions in obese mice. Our results are also consistent with studies that evaluated the effect of supplementation with a strain of bacteria belonging to the *Clostridiaceae* family (*Clostridium butyricum*), which not only showed enhanced expression of tight junction proteins but decreased colon inflammation in obese mice and in a mouse model of induced colitis (47,48).

Concerning *Lactobacillaceae* family members, the study conducted by Ahmadi et al. (8), demonstrated that supplementation with some strains of *Lactobacillus* and *Enterococcus* can increase taurine levels. As a result, leaky gut is reduced by stimulating tight junction expression in the colon, and inflammation is improved. Similarly, Wong et al. (49) observed an improvement in colon inflammation by *Lactobacillus casei* supplementation, via increasing taurine-conjugated bile acids. In this regard, this study showed that the *Lactobacillaceae* family, which decreased with age, had a positive correlation with tight junction expression and a negative association with inflammation genes. In addition, *Ligilactobacillus* is a genus member of the *Lactobacillaceae* family that has gained recognition for its health benefits for the host. In this sense, our analysis revealed a negative correlation between this genus and proinflammatory cytokines. Moreover, Yao et al. (50) examined the effect of *Ligilactobacillus salivaris* on colon inflammation and determined that this strain could reduce inflammatory cytokines and increase antiinflammatory ones. Thus, maintaining adequate levels of this family may prove beneficial in preventing age-related health decline.

Despite the limited knowledge of *Monoglobaceae*, it has been shown that *Monoglobus pectinilyticus*, a member of this family, can degrade pectin, which may reduce the severity of colitis (51). Additionally, pectin degradation appears to be capable of modulating intestinal immunity by increasing the expression of IgA (52). This could explain the association of the *Monoglobaceae* family with a better immune response found in the present study. The immune response of older adults may be affected as a result of the decline in the abundance of *Monoglobaceae* bacteria.

The present study indicated that the *Akkermansia* proportion increased with age. This rise was associated with the high expression of gut inflammation markers and the suppression of immune-related genes in the colon. Our findings are consistent with those observed by Van der Lugt et al. (16) who observed that *Akkermansia muciniphila* supplementation resulted in the downregulation of *Cd4* gene expression in aging mice. It has been observed in other studies that *Akkermansia* levels increase among centenarians (10,11), suggesting that these microorganisms may contribute to longevity, although there is not enough evidence to clarify their role in the maintenance of health.

This study provides insight into the diversity of gut microbiota composition as well as the relationship between the major hallmarks of aging and their association with the different bacteria found in the gut at different stages of life. The main limitation of this study was that only female mice were used in the experiment. However, several previous studies have been carried out only on male mice, which allows discussion and comparison with them. As an example, one research project using male mice found the same result, the decline in *Lactobacillus* and *Bacteroides* abundance by aging, and on the contrary, they found that *Akkermansia* abundance was higher in young mice (4). Further research is needed to confirm if this difference could be attributed to gender.

In summary, the older mice exhibited more gut microbiota diversity, but this was not translated into better health. On the contrary, old mice were losing levels of mutualist bacteria and increased the pathogens, which can contribute to triggering age-related diseases such as immunosenescence, inflammaging, and leaky gut. More investigations are necessary to establish the adequate level of each bacterium in order to find a new therapy that contributes to improving the quality of life in the older population.

## Supplementary Material

glae045_suppl_Supplementary_Tables_S1

## References

[1] World Health Organization. Ageing and Health. Published 2022. Accessed December 7, 2022. https://www.who.int/news-room/fact-sheets/detail/ageing-and-health

[2] Funk MC, Zhou J, Boutros M. Ageing, metabolism and the intestine. EMBO Rep. 2020;21(7):1–22. 10.15252/embr.202050047 PMC7332987 32567155

[3] Branca JJV, Gulisano M, Nicoletti C. Intestinal epithelial barrier functions in ageing. Ageing Res Rev. 2019;54:100938. 10.1016/j.arr.2019.100938 31369869

[4] Liu A, Lv H, Wang H, Yang H, Li Y, Qian J. Aging increases the severity of colitis and the related changes to the gut barrier and gut microbiota in humans and mice. J Gerontol A Biol Sci Med Sci. 2020;75(7):1284–1292. 10.1093/gerona/glz263 32048723

[5] Walrath T, Dyamenahalli KU, Hulsebus HJ, et al. Age-related changes in intestinal immunity and the microbiome. J Leukoc Biol. 2021;109(6):1045–1061. 10.1002/JLB.3RI0620-405RR 33020981 PMC8139861

[6] Franceschi C, Campisi J. Chronic inflammation (Inflammaging) and its potential contribution to age-associated diseases. J Gerontol A Biol Sci Med Sci. 2014;69:S4–S9. 10.1093/gerona/glu057 24833586

[7] Allam-Ndoul B, Castonguay-Paradis S, Veilleux A. Gut microbiota and intestinal trans-epithelial permeability. Int J Mol Sci. 2020;21(17):6402–6414. 10.3390/ijms21176402 32899147 PMC7503654

[8] Ahmadi S, Wang S, Nagpal R, et al. A human-origin probiotic cocktail ameliorates aging-related leaky gut and inflammation via modulating the microbiota/taurine/tight junction axis. JCI Insight. 2020;5(9):e132055. 10.1172/jci.insight.132055 32302292 PMC7253024

[9] Vaiserman AM, Koliada AK, Marotta F. Gut microbiota: a player in aging and a target for anti-aging intervention. Ageing Res Rev. 2017;35:36–45. 10.1016/j.arr.2017.01.001 28109835

[10] Bárcena C, Valdés-Mas R, Mayoral P, et al. Healthspan and lifespan extension by fecal microbiota transplantation into progeroid mice. Nat Med. 2019;25(8):1234–1242. 10.1038/s41591-019-0504-5 31332389

[11] Ragonnaud E, Biragyn A. Gut microbiota as the key controllers of “healthy” aging of elderly people. Immun Ageing. 2021;18(1):1–11. 10.1186/s12979-020-00213-w 33397404 PMC7784378

[12] Cao L, Lee SG, Melough MM, et al. Long-term blackcurrant supplementation modified gut microbiome profiles in mice in an age- dependent manner: an exploratory study. Nutrients. 2020;12(2):290. 10.3390/nu12020290 31973241 PMC7070352

[13] Brunt VE, Gioscia-Ryan RA, Richey JJ, et al. Suppression of the gut microbiome ameliorates age-related arterial dysfunction and oxidative stress in mice. J Physiol. 2019;597(9):2361–2378. 10.1113/JP277336 30714619 PMC6487935

[14] Luo D, Chen K, Li J, et al. Gut microbiota combined with metabolomics reveals the metabolic profile of the normal aging process and the anti-aging effect of FuFang Zhenshu TiaoZhi(FTZ) in mice. Biomed Pharmacother. 2020;121:109550. 10.1016/j.biopha.2019.109550 31704617

[15] Félix-Soriano E, Sáinz N, Gil-Iturbe E, et al. Changes in brown adipose tissue lipid mediator signatures with aging, obesity, and DHA supplementation in female mice. FASEB J. 2021;35(6):e21592. 10.1096/fj.202002531R 33960028 PMC12315960

[16] Van Der Lugt B, Van Beek AA, Aalvink S, et al. Akkermansia muciniphila ameliorates the age-related decline in colonic mucus thickness and attenuates immune activation in accelerated aging Ercc1 -/Δ7 mice. Immun Ageing. 2019;16(1):1–17. 10.1186/s12979-019-0145-z 30899315 PMC6408808

[17] Özkurt E, Fritscher J, Soranzo N, et al. LotuS2: an ultrafast and highly accurate tool for amplicon sequencing analysis. Microbiome. 2022;10(1):176. 10.1186/s40168-022-01365-1 36258257 PMC9580208

[18] Edgar RC. UPARSE: highly accurate OTU sequences from microbial amplicon reads. Nat Methods. 2013;10(10):996–998. 10.1038/nmeth.2604 23955772

[19] Bedarf JR, Beraza N, Khazneh H, et al. Much ado about nothing? Off-target amplification can lead to false-positive bacterial brain microbiome detection in healthy and Parkinson’s disease individuals. Microbiome. 2021;9(1):75. 10.1186/s40168-021-01012-1 33771222 PMC8004470

[20] Hauswedell H, Singer J, Reinert K. Lambda: The local aligner for massive biological data. Bioinformatics. 2014;30(17):i349–i355. 10.1093/bioinformatics/btu439 25161219 PMC4147892

[21] Yilmaz P, Parfrey LW, Yarza P, et al. The SILVA and “all-species Living Tree Project (LTP)” taxonomic frameworks. Nucleic Acids Res. 2014;42(D1):D643–D648. 10.1093/nar/gkt1209 24293649 PMC3965112

[22] Nikolova VL, Hall MRB, Hall LJ, Cleare AJ, Stone JM, Young AH. Perturbations in gut microbiota composition in psychiatric disorders: a review and meta-analysis. JAMA Psychiatry. 2021;78(12):1343–1354. 10.1001/jamapsychiatry.2021.2573 34524405 PMC8444066

[23] Cooper S, Levy I, Ben-Zvi H, et al. Flavobacteriaceae bacteremia in children: a multicenter study. Pediatr Infect Dis J. 2019;38(11):1096–1099. 10.1097/INF.0000000000002449 31469778

[24] Wu TR, Lin CS, Chang CJ, et al. Gut commensal *Parabacteroides goldsteinii* plays a predominant role in the anti-obesity effects of polysaccharides isolated from Hirsutella sinensis. Gut. 2019;68(2):248–262. 10.1136/gutjnl-2017-315458 30007918

[25] Wu S, Zhang Y, Ma J, et al. Interleukin-6 absence triggers intestinal microbiota dysbiosis and mucosal immunity in mice. Cytokine. 2022;153:155841. 10.1016/j.cyto.2022.155841 35276634

[26] Suzuki T. Regulation of intestinal epithelial permeability by tight junctions. Cell Mol Life Sci. 2013;70(4):631–659. 10.1007/s00018-012-1070-x 22782113 PMC11113843

[27] Luciano-Mateo F, Cabré N, Baiges-Gaya G, et al. Systemic overexpression of C-C motif chemokine ligand 2 promotes metabolic dysregulation and premature death in mice with accelerated aging. Aging (Milano). 2020;12(20):20001–20023. 10.18632/aging.104154 PMC7655213 33104522

[28] Siu G. Linking cd4 gene expression and T Cell Development. Curr Mol Med. 2001;1(5):523–532. 10.2174/1566524013363465 11899228

[29] Akatsu C, Shinagawa K, Numoto N, et al. CD72 negatively regulates b lymphocyte responses to the lupus-related endogenous toll-like receptor 7 ligand Sm/RNP. J Exp Med. 2016;213(12):2691–2706. 10.1084/jem.20160560 27810925 PMC5110020

[30] Abreu MT. Toll-like receptor signalling in the intestinal epithelium: how bacterial recognition shapes intestinal function. Nat Rev Immunol. 2010;10(2):131–144. 10.1038/nri2707 20098461

[31] Hansen GH, Rasmussen K, Niels-Christiansen LL, Danielsen EM. Lipopolysaccharide-binding protein: localization in secretory granules of Paneth cells in the mouse small intestine. Histochem Cell Biol. 2009;131(6):727–732. 10.1007/s00418-009-0572-6 19234712

[32] Ling Z, Liu X, Cheng Y, Yan X, Wu S. Gut microbiota and aging. Crit Rev Food Sci Nutr. 2022;62(13):3509–3534. 10.1080/10408398.2020.1867054 33377391

[33] Chen Y, Zhou J, Wang L. Role and mechanism of gut microbiota in human disease. Front Cell Infect Microbiol. 2021;11:625913. 10.3389/fcimb.2021.625913 33816335 PMC8010197

[34] Tran SMS, Hasan Mohajeri M. The role of gut bacterial metabolites in brain development, aging and disease. Nutrients. 2021;13(3):1–41. 10.3390/nu13030732 PMC7996516 33669008

[35] Sovran B, Hugenholtz F, Elderman M, et al. Age-associated impairment of the mucus barrier function is associated with profound changes in microbiota and immunity. Sci Rep. 2019;9(1):1–13. 10.1038/s41598-018-35228-3 30723224 PMC6363726

[36] Wu CS, Muthyala SDV, Klemashevich C, et al. Age-dependent remodeling of gut microbiome and host serum metabolome in mice. Aging (Milano). 2021;13(5):6330–6345. 10.18632/aging.202525 PMC7993679 33612480

[37] Hu C, Rzymski P. Non-photosynthetic melainabacteria (Cyanobacteria) in human gut: characteristics and association with health. Life. 2022;12(4):476. 10.3390/life12040476 35454968 PMC9029806

[38] Rizzatti G, Lopetuso LR, Gibiino G, Binda C, Gasbarrini A. Proteobacteria: A common factor in human diseases. Biomed Res Int. 2017;2017:9351507. 10.1155/2017/9351507 29230419 PMC5688358

[39] Chopyk DM, Grakoui A. Contribution of the intestinal microbiome and gut barrier to hepatic disorders. Gastroenterology. 2020;159(3):849–863. 10.1053/j.gastro.2020.04.077 32569766 PMC7502510

[40] Qiu W, Liu T, Liu X, et al. Enrofloxacin induces intestinal microbiota-mediated immunosuppression in zebrafish. Environ Sci Technol. 2022;56(12):8428–8437. 10.1021/acs.est.1c08712 35545936 PMC9228068

[41] Mazmanian SK, Kasper DL. The love-hate relationship between bacterial polysaccharides and the host immune system. Nat Rev Immunol. 2006;6(11):849–858. 10.1038/nri1956 17024229

[42] Pan M, Barua N, Ip M. Mucin-degrading gut commensals isolated from healthy faecal donor suppress intestinal epithelial inflammation and regulate tight junction barrier function. Front Immunol. 2022;13:1021094. 10.3389/fimmu.2022.1021094 36311778 PMC9597641

[43] Cui Y, Zhang L, Wang X, et al. Roles of intestinal Parabacteroides in human health and diseases. FEMS Microbiol Lett. 2022;369(1):fnac072. 10.1093/femsle/fnac072 35945336

[44] Neyrinck AM, Etxeberria U, Taminiau B, et al. Rhubarb extract prevents hepatic inflammation induced by acute alcohol intake, an effect related to the modulation of the gut microbiota. Mol Nutr Food Res. 2017;61(1):1500899. 10.1002/mnfr.201500899 26990039

[45] Guo Y, Zhu G, Wang F, et al. Distinct serum and fecal metabolite profiles linking with gut microbiome in older adults with frailty. Front Med (Lausanne). 2022;9:827174. 10.3389/fmed.2022.827174 35479954 PMC9035822

[46] Fang W, Xue H, Chen X, Chen K, Ling W. Supplementation with sodium butyrate modulates the composition of the gut microbiota and ameliorates high-fat diet-induced obesity in mice. J Nutr. 2019;149(5):747–754. 10.1093/jn/nxy324 31004166

[47] Liu M, Xie W, Wan X, Deng T. *Clostridium butyricum* protects intestinal barrier function via upregulation of tight junction proteins and activation of the Akt/mTOR signaling pathway in a mouse model of dextran sodium sulfate-induced colitis. Exp Ther Med. 2020;20(5):1–1. 10.3892/etm.2020.9138 32934675 PMC7471846

[48] Shang H, Sun J, Chen YQ. *Clostridium butyricum* CGMCC0313.1 modulates lipid profile, insulin resistance and colon homeostasis in obese mice. PLoS One. 2016;11(4):e0154373. 10.1371/journal.pone.0154373 27123997 PMC4849746

[49] Wong WY, Chan BD, Sham TT, et al. *Lactobacillus casei* strain shirota ameliorates dextran sulfate sodium-induced colitis in mice by increasing taurine-conjugated bile acids and inhibiting NF-κB signaling via stabilization of IκBα. Front Nutr. 2022;9:816836. 10.3389/fnut.2022.816836 35529468 PMC9069136

[50] Yao M, Lu Y, Zhang T, et al. Improved functionality of *Ligilactobacillus salivarius* Li01 in alleviating colonic inflammation by layer-by-layer microencapsulation. NPJ Biofilms Microbiomes. 2021;7(1):58. 10.1038/s41522-021-00228-1 34244520 PMC8270932

[51] Kim CC, Healey GR, Kelly WJ, et al. Genomic insights from *Monoglobus pectinilyticus*: a pectin-degrading specialist bacterium in the human colon. ISME J. 2019;13(6):1437–1456. 10.1038/s41396-019-0363-6 30728469 PMC6776006

[52] Lee HB, Kim YS, Park HY. Pectic polysaccharides: targeting gut microbiota in obesity and intestinal health. Carbohydr Polym. 2022;287:119363. 10.1016/j.carbpol.2022.119363 35422307

