# Supplementary material for: Intestinal Permeability, Gut Inflammation, and Gut Immune System Response Are Linked to Aging-Related Changes in Gut Microbiota Composition: A Study in Female Mice
Source: J Gerontol A Biol Sci Med Sci. 2024 Feb 14;79(4):glae045. doi: 10.1093/gerona/glae045 (PMC10957128; doi:10.1093/gerona/glae045)
Supplement: glae045_suppl_Supplementary_Tables_S1 [file glae045_suppl_supplementary_tables_s1.docx]

| **Table S1.** Primer sequencing | | |
| --- | --- | --- |
| **Gen** | **Forward (5’ → 3’)** | **Reverse (5’ → 3’)** |
| *Cldn2* | CAACTGGTGGGCTACATCCTA | CCCTTGGAAAAGCCAACCG |
| *Cldn8* | GCAACCTACGCTCTTCAAATGG | TTCCCAGCGGTTCTCAAACAC |
| *Cd4* | TCCTAGCTGTCACTCAAGGGA | TCAGAGAACTTCCAGGTGAAGA |
| *Cd72* | GCTCAGGGAGAAGATAAGTCAGC | GCGTCCTCGTGAGTCCTCT |
| *Tlr4* | TGGTTGCAGAAAATGCAGG | AGGAACTACCTCTATGCAGGG |
| *Tlr7* | CACCACCAATCTTACCCTTACC | CAGATGGTTCAGCCTACGGAA |
| *Tlr12* | TTGGAAGTTGTACCTCGGACT | GAAGTTGGGTAAGGTGCAGAC |
| *Ccl2* | AGCACCAGCCAACTCTCACT | TCATTGGGATCATCTTGCTG |
| *Ccl12* | ATTTCCACACTTCTATGCCTCCT | ATCCAGTATGGTCCTGAAGATCA |
| *Tjp1* | GCCGCTAAGAGCACAGCAA | TCCCCACTCTGAAAATGAGGA |
| *Tjp2* | ATGGGAGCAGTACACCGTGA | TGACCACCCTGTCATTTTCTTG |
| *Jam2* | GATCGTCGCCCTGGACTATC | GTGACTTCTTGACGGTGGTCT |
| *Lbp* | GATCACCGACAAGGGCCTG | GGCTATGAAACTCGTACTGCC |
| *Tnf-a* | CATCTTCTCAAAATTCGAGTGACAA | TGGGAGTAGACAAGGTACAACCC |
| *Il-10* | AAGGCAGTGGAGCAGGTGAA | CCAGCAGACTCAATACACAC |
| *Il-6* | GAGGATACCACTCCCAACAGACC | AAGTGCATCATCGTTGTTCATACA |
| *Gapdh* | TGTGTCCGTCGTGGATCTGA | CCTGCTTCACCACCTTCTTGAT |
